# The Physician's Visiting List for 1879

**Published:** 1878-11

**Authors:** 


### BIBLIOGRAPHICAL.

*The Physician's Visiting List for* 1879. Publishers—Lindsay & Blackiston, Philadelphia.

The Twenty-Eighth edition of this popular and useful appointment book is at hand, and is as attractive and interesting as ever. It makes a very admirable dental appointment book, and is of convenient size and arrangement.

